# A Rab-bit hole: Rab40 GTPases as new regulators of the actin cytoskeleton and cell migration

**DOI:** 10.3389/fcell.2023.1268922

**Published:** 2023-09-06

**Authors:** Andrew J. Neumann, Rytis Prekeris

**Affiliations:** Department of Cell and Developmental Biology, School of Medicine, University of Colorado Anschutz Medical Campus, Aurora, CO, United States

**Keywords:** Rab40 GTPases, SOCS box, ubiquitination, actin, cytoskeleton, cell migration, Rho GTPases

## Abstract

The regulation of machinery involved in cell migration is vital to the maintenance of proper organism function. When migration is dysregulated, a variety of phenotypes ranging from developmental disorders to cancer metastasis can occur. One of the primary structures involved in cell migration is the actin cytoskeleton. Actin assembly and disassembly form a variety of dynamic structures which provide the pushing and contractile forces necessary for cells to properly migrate. As such, actin dynamics are tightly regulated. Classically, the Rho family of GTPases are considered the major regulators of the actin cytoskeleton during cell migration. Together, this family establishes polarity in the migrating cell by stimulating the formation of various actin structures in specific cellular locations. However, while the Rho GTPases are acknowledged as the core machinery regulating actin dynamics and cell migration, a variety of other proteins have become established as modulators of actin structures and cell migration. One such group of proteins is the Rab40 family of GTPases, an evolutionarily and functionally unique family of Rabs. Rab40 originated as a single protein in the bilaterians and, through multiple duplication events, expanded to a four-protein family in higher primates. Furthermore, unlike other members of the Rab family, Rab40 proteins contain a C-terminally located suppressor of cytokine signaling (SOCS) box domain. Through the SOCS box, Rab40 proteins interact with Cullin5 to form an E3 ubiquitin ligase complex. As a member of this complex, Rab40 ubiquitinates its effectors, controlling their degradation, localization, and activation. Because substrates of the Rab40/Cullin5 complex can play a role in regulating actin structures and cell migration, the Rab40 family of proteins has recently emerged as unique modulators of cell migration machinery.

## 1 Introduction

Cell migration is an essential process for the function and maintenance of complex biological processes in all eukaryotes, from single cell protists to complex multicellular organisms. Subsequently, control of migratory machinery must be maintained as improper or dysregulated migration can result in developmental defects and cancer metastasis (Franz et al., 2002; Bravo-Cordero et al., 2012; Trepat et al., 2012). One factor tightly regulated during the migratory process is the actin cytoskeleton. Actin filaments dynamically form a variety of structures which provide the forces necessary for cells to undergo locomotion (Schaks et al., 2019; Lappalainen et al., 2022). Highly dynamic formation and breakdown of these structures in response to extracellular signals are generally considered to be under the spatial and temporal control of the Rho family of GTPases (Lawson and Ridley, 2017). Many proteins modulate Rho GTPase activity. Furthermore, other proteins outside of Rho GTPases can also directly modulate the actin cytoskeleton, resulting in a multitude of networks by which actin dynamics and cell migration can be regulated. Here we will discuss the actin structures involved in cell migration, how Rho GTPases provide canonical control over these actin structures, and the role of Rab40 GTPases in modulating actin structures. The family of Rab40 GTPases has recently been identified as regulators of actin dynamics during cell migration and belong to an evolutionarily unique family of Rabs that use ubiquitination to exhibit control over the degradation, activation, and spatiotemporal localization of various actin regulators, thus modulating cell migration.

## 2 The role of actin structures in cell migration

**FIGURE 1:**
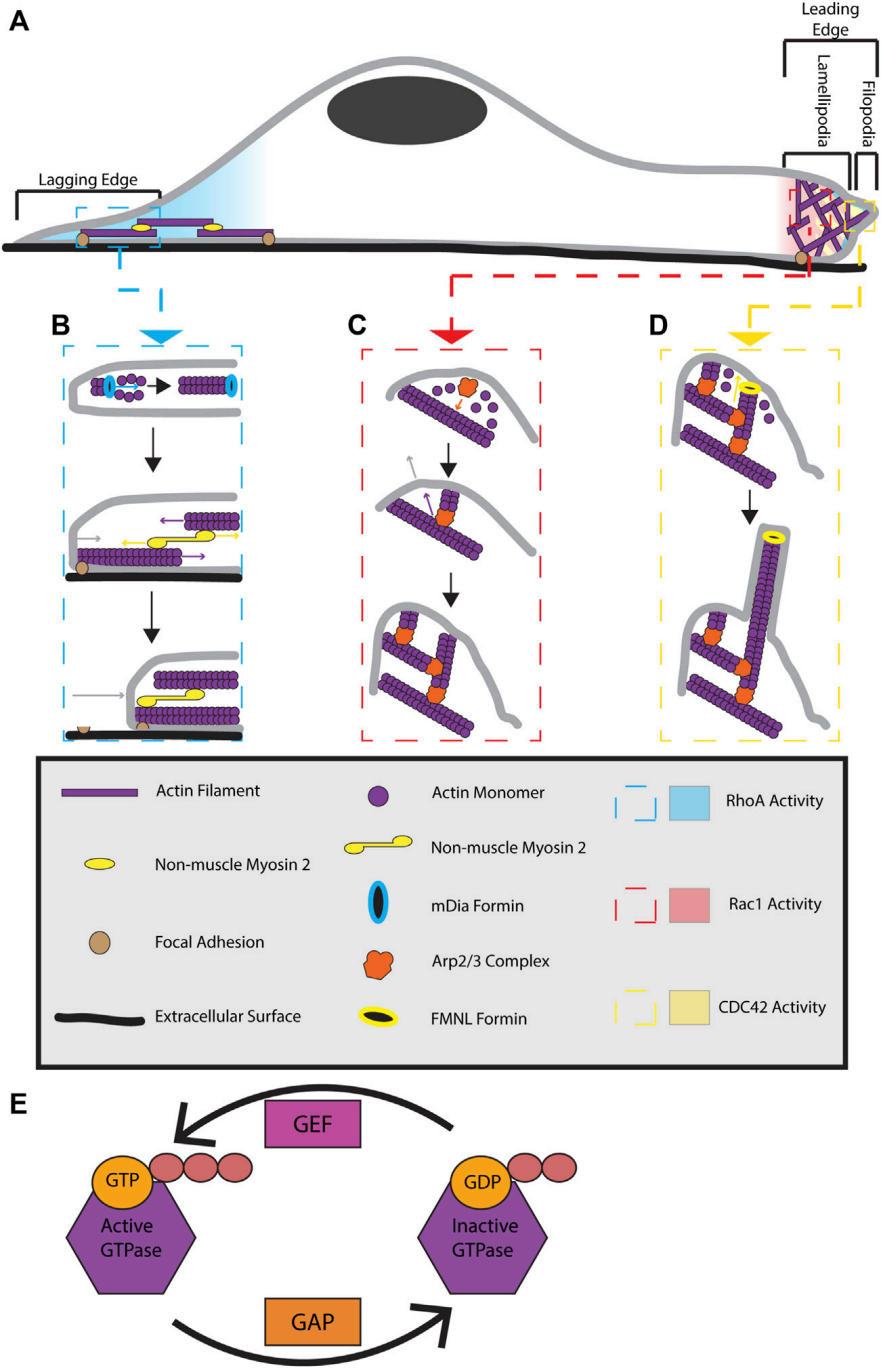
Formation and regulation of actin cytoskeletal structures during cell migration. **(A)** Migrating cells display distinct polarization. At the lagging end of the cell, RhoA is active and stimulates the formation of actin-myosin stress fibers. At the leading edge of the cell RhoA, Cdc42, and Rac1 are active. RhoA activity is localized to the very front edge of a migrating cell where it is proposed to stimulate leading edge ruffling. Cdc42 activity is at the front of the leading edge and stimulates filopodia formation. Rac1 activity is present throughout the whole leading edge and contributes to lamellipodia extension. **(B)** At the lagging edge, RhoA activates mDIA formins to make linear actin filaments. These filaments are joined together with alternating polarity by non-muscle myosin 2 motors to form stress fibers. Stress fibers are anchored to the cell and extracellular surface by focal adhesions. Myosin 2 activity pulls the actin filaments in stress fibers together, providing a contractile force on the rear of the cell. The contractile force breaks the connection between focal adhesions located at the rear of the cell and the extracellular surface. This results in stress fiber contraction pulling the lagging end of the cell forward, causing cell body translocation. **(C)** At the leading edge, Rac1 activity triggers lamellipodia extension by activating WRC (not pictured). WRC activates the Arp2/3 complex, allowing it to bind previously formed actin filaments. Actin monomers use the Arp2/3 complex as a site of nucleation, allowing a new actin filament to polymerize from roughly a 70⁰ angle to that of the previously existing filament. Continuous Arp2/3 complex induced branched actin polymerization results in a pushing force being applied to a large area of the membrane. This causes the membrane to extend, forming a lamellipodia. **(D)** Cdc42 activity at the leading edge can activate FMNL formins. FMNL formins bind to free barbed ends of branched actin filaments, causing the linear extension of that actin filament. These linear actin filaments provide a pushing force on a small surface area of the membrane, resulting in skinny membrane protrusions known as filopodia. The coordination of all these actin cytoskeletal structures provides the forces necessary for cellular locomotion. **(E)** GTPases are active when bound to GTP and inactive when GDP bound. GAPs function to inactive GTPases by converting GTP to GDP while GEFs activate GTPases by replacing GDP with GTP.

The key to highly dynamic changes in the actin cytoskeleton is constant and highly regulated assembly and disassembly of actin filaments. The majority of cytosolic actin exists as a monomer (G-actin) that is bound to either ADP or ATP nucleotides. Usually, ATP-bound G-actin is preferentially incorporated into a growing actin filament at the filament’s barbed end. However, this can change at low concentrations of monomeric G-actin. As actin filaments grow, ATP is hydrolyzed into ADP, and ADP-bound actin then spontaneously dissociates from the actin filament at the filament’s pointed end. These constant addition and dissociation events of actin monomer to an actin filament provide the basic mechanics for actin dynamics and treadmilling, leading to highly dynamic actin microfilaments. Since the molecular machinery governing actin dynamics, polymerization, depolymerization, and rearrangement have been recently described in several excellent reviews (Rottner et al., 2017; Schaks et al., 2019; Lappalainen et al., 2022), here we will only briefly discuss actin filament formation during cell migration and the how these actin filaments function to polarize the cell and allow for cellular locomotion (Figure 1A).

### 2.1 Branched actin

Branched actin filament polymerization is controlled by the Actin Related Protein (Arp) 2/3 complex. The Arp2/3 complex binds actin filaments near the pointed end and nucleates the polymerization of a new actin filament at an angle roughly 70° to the original filament (Mullins et al., 1998). During cell migration, branched actin filaments are generally localized to the leading edge of a cell where they generate a pushing force against the plasma membrane, resulting in extension of the leading edge and formation of a structure known as the lamellipodia (Figure 1C). Increased pushing force on the leading edge membrane is accomplished by an increase of branched actin density and polymerization in the lamellipodia (Bisi et al., 2013; Li et al., 2022). Thus, branched actin polymerization controls lamellipodia extension and retraction.

Cells migrate in the direction of branched actin polymerization and lamellipodia extension. Cells contain multiple Arp2/3 complex regulators which function to control migration and lamellipodia formation (Molinie and Gautreau, 2017). Local activity of these regulators control Arp2/3 mediated branched actin polymerization and, in turn, control the direction of cell migration. For example, upon local inhibition of the Arp2/3 complex, lamellipodia and branched actin networks deform, allowing branched actin polymerization and lamellipodia formation to begin in another area of the cell. The cell then turns and begins migrating in the direction of the newly polymerizing branched actin network (Gorelik and Gautreau, 2015; Simanov et al., 2021; Fregoso et al., 2022). Furthermore, branched actin polymerization is essential to lamellipodia formation as fibroblasts without functional Arp2/3 complex do not form lamellipodia. Without a lamellipodia, cells are able to undergo random cell migration, however, they exhibit no persistence in directionality of movement (Suraneni et al., 2012). Accordingly, branched actin networks are a vital structure for directional cell migration and lamellipodia dynamics.

Besides lamellipodia, invadopodia and podosomes are other structures reliant on branched actin networks. Invadopodia and podosomes are membrane protrusions that form during cell migration through the three-dimensional extracellular matrix (3D ECM). The base of these protrusions is formed from an Arp2/3 dependent branched actin network. Further actin polymerization from the barbed ends of the branched actin base results in the protrusion and maturation of the invadopodia or podosome (Yamaguchi et al., 2005; Albiges-Rizo et al., 2009). Matrix metalloproteinases (MMPs) are released from the ends of mature invadopodia and podosomes. MMPs function to cause the local remodeling of extracellular material, creating a path through the 3D ECM for the migrating cell. Thus, through the action of MMPs, invadopodia and podosomes assist cellular invasion and migration through a 3D ECM (Chen, 1989; Buccione et al., 2004).

### 2.2 Stress fibers

Linear actin filaments are nucleated and polymerized by a family of proteins known as formins (Chesarone et al., 2010). Non-muscle myosin 2 motors bundle together with polymerized linear actin filaments to form an actin myosin bundle. Bundles with alternating actin polarity are joined together to form a stress fiber (Cramer et al., 1997; Hotulainen and Lappalainen, 2006; Naumanen et al., 2008). Three main types of stress fibers can be formed: dorsal stress fibers, transverse arcs, and ventral stress fibers. While all types of stress fibers are important for cell migration, ventral stress fibers (further referred to as stress fibers) provide the function of interest for this review and will be the focus of this section.

Stress fibers are located along the ventral (bottom) side of the cell, run from the leading to lagging end of a cell, and make connections to focal adhesion sites at both ends of the fiber (Hotulainen and Lappalainen, 2006; Naumanen et al., 2008). In actin-myosin bundles, myosin 2 activity pulls antipolar actin filaments together, providing a contractile force. Through the connections of stress fibers to the ventral cellular surface, this contractile force is transferred to the cell and pulls the lagging edge of the cell towards the leading edge, contributing to cell body translocation (Figure 1B) (Svitkina, 2018).

The contractile force provided by stress fibers is essential for standard cell body translocation during cellular locomotion. Inhibition of non-muscle myosin 2 activity with blebbistatin prevents standard retraction of the lagging end of the cell body after leading end extension, resulting in the lagging end of the cell trailing behind during migration. Interestingly, while disrupting standard cellular locomotion, blebbistatin has varying effects on the velocity and directionality of cell migration (Kolega, 2006; Zhang et al., 2017; Richter et al., 2021). Thus, while having varying effects on cell migration, the actin-myosin contractility found in stress fibers is essential to lagging end body translocation during cell migration.

### 2.3 Focal adhesions

Focal adhesions are a type of integrin adhesion complex which function to connect the actin cytoskeleton to the cell’s extracellular surface and are crucial for cell migration. Focal adhesions begin to form as clusters of α and β-integrin heterodimers located on the ventral surface of protruding cellular structures interact with the extracellular matrix (Wehrle-Haller, 2012; Mishra and Manavathi, 2021). Immature nascent focal adhesions typically begin to form towards the leading edge of a cell. As signals trigger focal adhesion maturation, various regulatory proteins, including actin-binding proteins, start accumulating at the focal adhesion sites, allowing for the strengthening of stress fiber attachment (Bachir et al., 2014; Mishra and Manavathi, 2021).

During cell migration, focal adhesions allow for the contractile force generated by stress fibers to be transmitted to the cell body and extracellular surface. Because stress fibers are connected on both ends to leading and lagging end focal adhesions, without focal adhesion regulation, the contractile force achieved by stress fibers would pull both leading and lagging edges towards the cell center, preventing locomotion. Accordingly, cell body translocation requires focal adhesion modulation. Actin-myosin contractility increases protein accumulation to focal adhesions, strengthening the connection between these focal adhesions and the extracellular surface (Wolfenson et al., 2011). During migration, more surface area and focal adhesions are localized to the cells leading edge. This increases leading edge adhesion strength to the extracellular surface as compared to that of the lagging edge, increasing the traction force in the leading edge. Thus, contractile forces caused by stress fibers results in the strengthening of leading edge focal adhesions, release of lagging edge focal adhesions from the extracellular surface, lagging edge contraction, and cell body translocation (Chen, 1979; Kaverina et al., 2002; Svitkina, 2018). Focal adhesions accordingly provide the coupling of actin cytoskeletal forces and extracellular attachment during cell migration.

## 3 Rho GTPases as regulators of actin cytoskeletal structures during migration

While the previously discussed actin based cytoskeletal structures are important for cell migration, the assembly and disassembly of these structures must be spatially and temporally regulated in order for them to be productive towards the goal of cellular locomotion. The Rho GTPase family of proteins is widely considered as master regulators of actin dynamics and cell migration. Each member provides spatial and temporal control of the formation of the actin structures previously discussed. Like all small monomeric GTPases, these proteins are subject to activation by guanine exchange factors (GEFs) and deactivation by GTPase-activating proteins (GAPs) (Figure 1E) (Ridley, 2013). Although eukaryotic organisms can have up to 20 Rho family members, the 3 “classical” Rho GTPases; Rac1, Cdc42, and RhoA, provide broad regulation of the aforementioned actin cytoskeleton structures and will be the focus of this discussion (Lawson and Ridley, 2017). Importantly, each of these three GTPases are conserved in all eukaryotes and are clearly established as playing key roles in the coordination of cell polarity and migration. Here, we will briefly discuss mechanisms tying together these three Rho GTPases and their function as it relates to branched actin, stress fibers, and focal adhesions.

### 3.1 Rac1 GTPase

Rac1 signals locally through the Wiskott-Aldrich syndrome protein (WASP)-family verprolin-homologous protein (WAVE) regulatory complex (WRC) to activate the Arp2/3 complex and stimulate branched actin polymerization (Figure 1C) (Ridley, 2015; Schaks et al., 2019). In *Drosophila melanogaster* border cells, local Rac1 activation has been shown to be sufficient to cause local membrane ruffling, inducing formation of a leading edge and causing cells to migrate in the direction of Rac1 activation (Wang et al., 2010; Montell et al., 2012).

While Rac1 is localized along the plasma membrane and to the cell nucleus, FRET (Förster resonance energy transfer) biosensors have shown that active (GTP-bound) Rac1 is localized predominately at the leading edge of migrating cells. Specifically, active Rac1 is localized directly at the areas of membrane ruffles, indicating it is active at the site of branched actin polymerization (Kraynov et al., 2000; Fritz and Pertz, 2016). Interestingly, when stimulated with epidermal growth factor (EGF), Rac1 activity is seen to quickly increase at the leading edge. However, activity decreases over time after EGF treatment (Kurokawa et al., 2003). In contrast, Rac1-FRET biosensors in fibroblasts treated with platelet-derived growth factor (PDGF) show that Rac1 activity at the leading edge of a cell begins when lamellipodia start to extend and continues as lamellipodia extension stalls. Rac1 activity continues after PDGF treatment as lamellipodia retract, however, activity moves away from the leading edge (Martin et al., 2016). Thus, while Rac1 activity is spatially regulated specifically at the leading edge of migrating cells, its temporal regulation seems to vary based on migratory stimuli.

### 3.2 Cdc42 GTPase

Like Rac1, Cdc42 contributes to polarity establishment and lamellipodia formation at the leading edge of a cell (Nobes and Hall, 1999; Etienne-Manneville, 2004). Cdc42 is capable of activating N-WASP, which proceeds to activate the Arp2/3 complex, resulting in branched actin polymerization (Rohatgi et al., 1999). Furthermore, Cdc42 can also activate Rac1 (Nobes and Hall, 1995). Accordingly, it is generally accepted that Rac1 and Cdc42 act cooperatively to stimulate lamellipodia extension and cell migration.

Independent of Rac1 activity at the leading edge, Cdc42 can also activate the FMNL family of formins. These formins attach to the barbed ends of branched actin filaments nucleated by the Arp2/3 complex. FMNL-induced polymerization of these actin filaments causes the formation of filopodia, skinny membrane protrusions caused by the forces exhibited by linear actin on the plasma membrane (Figure 1D) (Block et al., 2012). The forces generated by Cdc42-activated FMNL formins are essential to lamellipodia protrusion velocity, lamellipodia width, and actin density inside the lamellipodia (Kage et al., 2017).

The use of Cdc42-FRET biosensors in PDGF stimulated fibroblasts has shown the temporal and spatial activation of Cdc42 during lamellipodia extension. Cdc42 is generally active at low levels at the leading edge prior to chemoattractant stimulation. Upon PDGF treatment, Cdc42 activity increases as a lamellipodia begins to extend out from the leading edge. Cdc42 activity continues after PDGF treatment for a short time as the lamellipodia retracts (Martin et al., 2016).

### 3.3 RhoA GTPase

RhoA is the Rho GTPase responsible for formation and maintenance of stress fibers and focal adhesions (Nobes and Hall, 1999). These responsibilities are accomplished through the interaction of RhoA with a variety of effectors. One of the main effectors of RhoA is the Rho associated coil-coil kinase (ROCK). ROCK activation increases myosin light chain 2 phosphorylation, causing increased contractility of stress fibers. ROCK activity also leads to inactivation of cofilin, an actin remodeling protein, thus increasing actin polymerization. Furthermore, ROCK also regulates focal adhesions turnover and maturation as fibroblasts without ROCK can have larger and immature focal adhesions (O'Connor and Chen, 2013; Julian and Olson, 2014). Regulation of stress fibers also occurs through RhoA activation of mDia, a formin known for its role in stress fiber formation (Figure 1B) (Watanabe et al., 1999). As such, RhoA plays a significant role in controlling the elements necessary for contractility at the lagging end of a migrating cell.

While RhoA is best known for its regulation of cell contractility at the lagging edge, it has been found that RhoA can be active at the leading edge of migrating fibroblasts. While it is not mechanistically known how, RhoA has been shown to be necessary in promoting membrane ruffling of the lamellipodia during cell migration (O'Connor et al., 2000; Kurokawa and Matsuda, 2005; O'Connor and Chen, 2013; Fritz and Pertz, 2016). RhoA-FRET biosensors show that RhoA activity at the leading edge of fibroblasts is constant until the addition of PDGF. During PDGF induced lamellipodia extension, RhoA activity diminishes, but does not disappear. Once lamellipodia extension stalls, RhoA activity levels rises to that of before extension and continues as the lamellipodia retracts (Martin et al., 2016).

### 3.4 Antagonistic interactions between RhoA and Rac1 during cell migration

The presence of both RhoA and Rac1 activity at the leading edge of a migrating cell presents a conceptual problem because these two GTPases positively regulate polymerization of structurally different actin filaments (linear vs. branched) that are often functionally in opposition to each other (contraction vs. pushing forces). Originally, it was generally believed that RhoA activity was lagging edge localized and only positively regulated actin myosin contractility, a function counter to that of Rac1. Accordingly, it was postulated that RhoA needs to be inactivated at the leading edge to allow for cell polarization and directional migration. However, the discovery that RhoA can promote actin ruffles in the lamellipodia suggested a synergy between RhoA and Rac1 activity. While debated, this observation has provided the initial framework necessary to begin rationalizing the coexistence of activated RhoA and Rac1 at the at leading edge (O'Connor et al., 2000; O'Connor and Chen, 2013).

Despite these findings, the general dogma is that RhoA and Rac1 activity, in most cases, is mutually exclusive and is thus spatially and temporally segregated during cell migration, with Rac1 being predominately active at the leading edge. This Rac1-RhoA polarity axis is believed to be responsible in determining the direction of cell migration and is maintained, in part, through antagonistic activity of Rac1 and RhoA and their effectors (Schaks et al., 2019). As examples, p21 activated kinase 1 (PAK1), a Rac1 effector, interacts with the RhoA GEF, GEF-H1. Upon interaction with PAK1, GEF-H1 is relocated away from leading edge localized RhoA and binds to microtubules, preventing it from activating RhoA at the leading edge (Zenke et al., 2004). Furthermore, active Rac1 also recruits and binds to p190bRhoGAP, a GAP for RhoA. Upon binding of Rac1, p190bRhoGAP binds to active RhoA and deactivates it, preventing RhoA activity at the leading edge (Bustos et al., 2008).

Conversely, RhoA can signal through ROCK to inhibit Rac1 activity. Active ROCK directly phosphorylates FilGAP, a Rac1-specific GAP. Phosphorylation of FilGAP results in the deactivation of Rac1 (Saito et al., 2012). ROCK also indirectly activates ARHGAP22, a Rac-GAP, through its modulation of actin myosin contractility. A ROCK induced increase in actin myosin contractility results in the activation of ARHGAP22 and the subsequent deactivation of Rac1 (Sanz-Moreno et al., 2008). Interestingly, BPGAP1 (also known as ARHGAP8), a leading edge localized scaffold, has been recently identified to promote antagonism of the Rac1-RhoA polarity axis in a manner where it does not encourage activity of its activator and deactivation of its competitor. Instead, BPGAP1 binds active RhoA. Upon active RhoA binding, BPGAP1 is able to bind inactive Rac1. BPGAP1 is then involved in the deactivation of RhoA and activation of Rac1. Thus, BPGAP1 presents itself as a unique coregulator of RhoA and Rac1 as it is activated by RhoA binding, but promotes Rac1 activity (Wong et al., 2023).

Taken together, this complicated system of Rac1 and RhoA antagonism, mediated through effectors and RhoA and Rac1 specific GAPs and GEFs, ensures spatiotemporal regulation of actin dynamics and cell migration while also providing insight into the complexity of proteins outside of the family of Rho GTPases that further regulate the actin cytoskeleton and cell migration. In line with such complexity, many other families of proteins have arisen as exerting control over actin polymerization. Continuing our discussion, we will focus on the Rab40 family of GTPases, unique proteins that have recently been discovered as regulators of actin dynamics and cell migration.

## 4 The Rab40 family as novel regulators of cell migration and actin dynamics

### 4.1 Rab GTPases and cell migration

Rab GTPases are well documented members of a large protein family which function as protein switches involved in membrane trafficking. In brief, Rabs are geranyl-geranylated, allowing them to, when active, associate with membranes. Typically, Rabs associate with and provide identity for vesicles and membrane coated organelles, functioning by recruiting a variety of effectors which regulate vesicle motility, docking, and fusion (Stenmark, 2009; Langemeyer et al., 2018; Homma et al., 2021). Through their function in membrane trafficking, a variety of Rabs have been implicated in cell migration. For example, Rab5, 5a, 10, 11b, 13, 21, 25, and 35 have all been implicated in the recycling and trafficking of integrins in a variety of cancer cells during cell migration, thus regulating focal adhesion turnover. Furthermore, Rab1b, 2a, 4, 5, 7, 8, 27a/b, and 37 have all been implicated in the membrane trafficking and secretion of MMPs and MMP inhibitors, thus regulating cell migration and invasion through the 3D ECM (Jin et al., 2021). Many Rab GTPases play a role in cell migration through regulation of membrane trafficking. However, the Rab40 family of proteins has recently emerged as a novel, yet minimally studied Rab family involved in actin cytoskeleton regulation and cell migration through a mechanism independent of canonical Rab membrane trafficking.

### 4.2 Origins of the Rab40 family

Recently, the Rab40 family of proteins has emerged as a novel, yet minimally studied protein family involved in actin cytoskeletal regulation. Evolutionarily, *Rab40* originated in the bilaterians as a result of a duplication event from *Rab18.* Duplication of *Rab40* occurred again during the early evolution of vertebrates, as two genes, *Rab40B* and *Rab40C* are present in genome of all vertebrates (Klöpper et al., 2012; Coppola et al., 2019; Duncan et al., 2021). *Rab40* duplication is again observed in higher primates with the emergence of *Rab40A* and *Rab40A-Like* (*Rab40AL*). Unlike *Rab40B* and *Rab40C, Rab40A*, and *Rab40AL* are both located on the X chromosome. Interestingly, the *Rab40AL* sequence contains no introns and is highly similar to the *Rab40A* exonic sequence. Accordingly, it has been proposed that *Rab40AL* arose as the result of a retrotranscription based duplication event of *Rab40A* (Pereira-Leal and Seabra, 2000; 2001; Lawson and Zhang, 2009; Coppola et al., 2019; Duncan et al., 2021).

**FIGURE 2:**
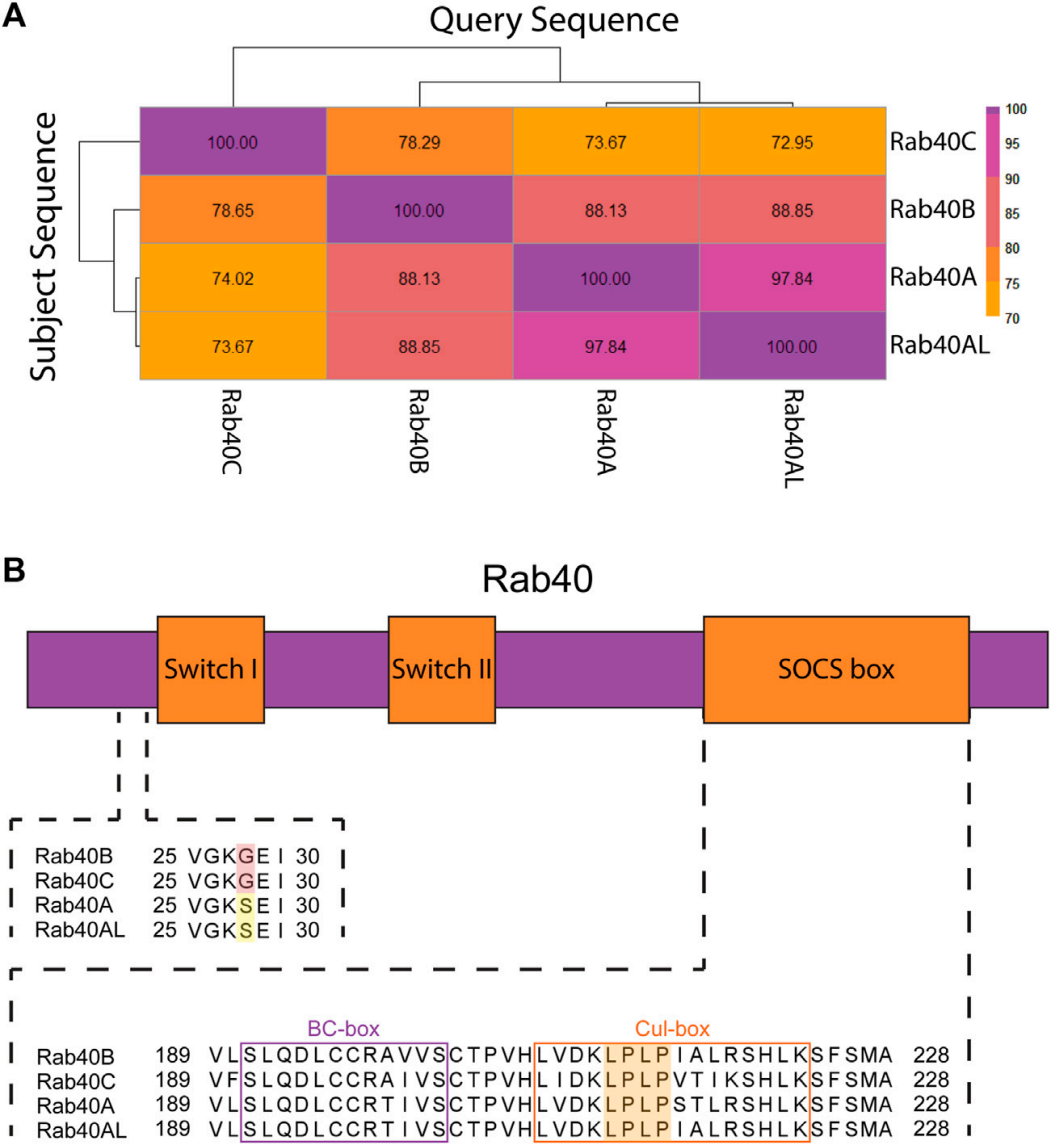
Protein structure of the Rab40 family. **(A)** Sequences of *Homo sapiens* Rab40 proteins were BLASTED against each other. Percent identity of the query sequence compared to the subject sequences was recorded and a heatmap was made using the Pheatmap function in R. **(B)** Diagram of Rab40 protein domains (not to scale) and alignment of regions of *Homo sapiens* Rab40 amino acid sequence. Upstream of Switch-I is an unexpected glycine residue in Rab40B and Rab40C (red highlight) involved in GEF binding that has reverted back to a serine residue in Rab40A and Rab40AL (yellow highlight). The SOCS box domain contains both a BC-box that facilitates Elongin B and C binding (purple outline) and a Cul-box that binds Cullin (orange outline). The LPLP motif in the Cul-box is conserved across all 4 Rab40 family members and creates specificity for Cullin5 binding (orange highlight).

Rab40 protein sequence replicates gene duplication events. Upon duplication from *Rab18,* Rab40 protein evolved a C-terminally located SOCS box domain, setting it apart from all other Rab GTPases. The subsequent duplication event, creating Rab40B and Rab40C, resulted in the most diversity within the Rab40 family. Human Rab40B and Rab40C are only about 78% similar. This is, in part, due to three glycine residues inserted C-terminally from the SOCS box domain that is unique to Rab40C as compared to the rest of the Rab40 family. The final duplication event, resulting in Rab40A and Rab40AL, produced two nearly (97%) identical proteins, the main differences occurring with Rab40A containing an arginine residue deletion at its C-terminal end. Interestingly, human Rab40A and Rab40AL are 88% similar to Rab40B, but are only about 72% similar to Rab40C (Figure 2A). This indicates that the event resulting in *Rab40A* and *Rab40AL* was from a duplication of *Rab40B*. The similarity between these Rab40 proteins seen across duplication events suggests that there may be a variety of overlapping functions between the members of the Rab40 family. Specifically, overlapping functions may be expected between duplication pairs, however, protein similarity suggests potential similar functions between Rab40A/Rab40AL and Rab40B as well.

### 4.3 Unique domains of the Rab40 family

Like other Rabs, the Rab40 family of proteins contain a conserved globular domain that houses the two switch regions found in Rab GTPases (Switch-I and Switch-II). As in all small monomeric GTPases, Rab binding to GTP stabilizes the two switch regions and locks the protein in an active conformation. Hydrolysis of GTP to GDP relaxes the switch regions, locking the protein in an inactive state (Pylypenko et al., 2018). Upstream of Switch-I is a highly conserved serine or threonine residue present in most Rabs that facilitate GEF binding. This residue can be mutated to glycine to generate GDP-bound Rabs that usually function as dominant-negative mutants by presumably sequestering Rab-specific GEFs that accumulate in non-productive GEF/Rab-GDP complexes. Interestingly, human Rab40B and Rab40C have naturally evolved a glycine residue at this location, raising the question of how Rab40-specific GEFs (not identified yet) may function to activate Rab40 family members. Even more surprisingly, Rab40A and Rab40AL have reverted to containing a serine residue at this site (Figure 2B) (Duncan et al., 2021). The variation of residues at this site in the Rab40 family suggests that the Rab40 family may have unique GTPase properties as compared to other Rabs.

As previously mentioned, the Rab40 family is unique among all other Rabs in that it contains a C-terminally located SOCS box domain. SOCS box domain containing proteins interact with a Cullin-RING E3 Ligase complex which functions to ubiquitinate its client proteins. The SOCS box domain was originally discovered through comparison of the cytokine inducible SH2-containing protein family. Protein sequence analysis of this family showed a conserved, 40 residue domain, that consisted of two highly conserved regions separated by a 2–10 residue variable region (Starr et al., 1997). The N-terminal of these two conserved regions is known as the BC-box. The BC-box recruits and binds the adaptors of the Cullin-RING E3 Ligase complex Elongin B and C. The C-terminal of the two conserved domains is the Cul-box which interacts with the Cullin family of proteins and provides specificity as to which Cullins participates in the Cullin-Ring E3 ligase complex (Figure 2B) (Linossi and Nicholson, 2012).

**FIGURE 3:**
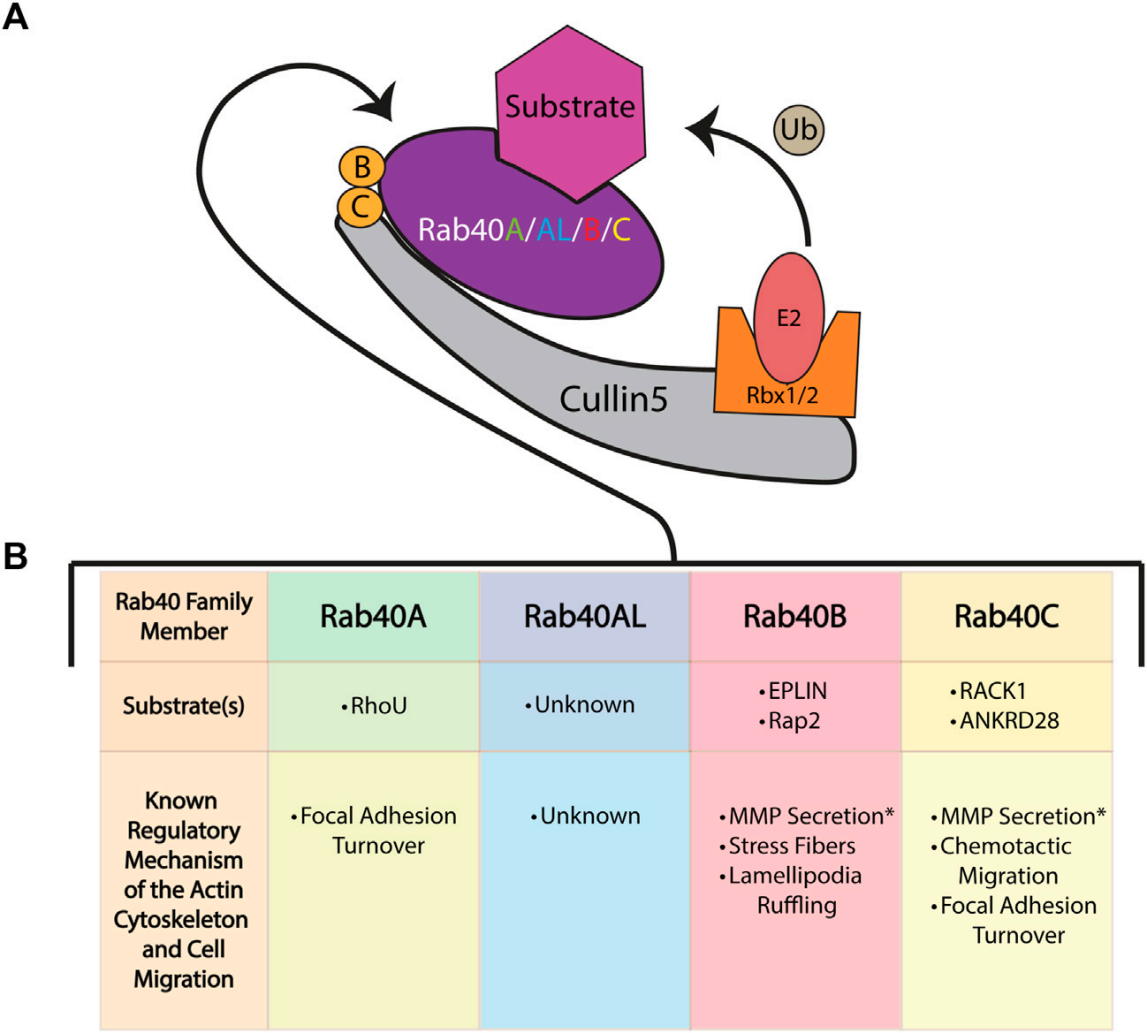
The role of the Rab40 family of proteins in cell migration and actin cytoskeleton regulation. **(A)** The Rab40 proteins bind to Cullin5 to function as part of a Cullin-RING E3 ubiquitin ligase complex. This complex consists of the scaffold protein Cullin5, a SOCS box containing protein (Rab40), the adaptor proteins Elongin B and Elongin C, and the RING protein Rbx1/2. The complex functions to ubiquitinate (Ub) its substrates. **(B)** The known mechanisms and substrates of the Rab40 family of proteins [colors correspond to Rab40 name in (A)] and the Cullin-RING E3 ubiquitin ligase complex as it relates to cell migration and the actin cytoskeleton. * Signifies a migration related function of Rab40 thought to be independent of the Cullin-RING E3 ubiquitin ligase complex.

Within the Cul-box is an LPφP motif (where φ is a hydrophobic residue) that is recognized as the primary sequence which determines the specificity of Cullin binding (Kim et al., 2013). The Rab40 family contains an LPLP sequence at this motif that is conserved across all bilaterians (Figure 2B) (Duncan et al., 2021). It was originally found that, in *Xenopus laevis*, XRab40 (the *Xenopus* Rab40c homolog) binds to XCullin5 (the *Xenopus* Cullin5 homolog) (Lee et al., 2007). Since then, human Rab40A, Rab40B, and Rab40C have all been confirmed as binding Cullin5 and forming a Cullin-RING E3 ubiquitin ligase complex (Figure 3A) (Dart et al., 2015; Yatsu et al., 2015; Day et al., 2018; Linklater et al., 2021; Duncan et al., 2022; Han et al., 2022). Further confirmation of the LPLP motif specifying Cullin5 binding has been accomplished through the mutation of the LPLP sequence to 4 alanine residues (SOCS 4A mutant) leading to drastically reduced Cullin5 binding to Rab40B and Rab40C (Duncan et al., 2021; Linklater et al., 2021; Duncan et al., 2022; Han et al., 2022). Rab40A binding to Cullin5 has not been tested with the SOCS 4A mutant.

The similarity in binding of Cullin5, conservation of domains, and extreme sequence homology among duplication pairs exhibited by the Rab40 family, suggests that these proteins act in a variety of redundant manners. However, study of the Rab40 family in relation to actin cytoskeleton regulation and cell migration has only recently begun and remains minimally investigated. Accordingly, work has mainly focused on individual Rab40 family members in specific cellular roles and has mainly failed to address the potential redundancy of function within the Rab40 family. Accordingly, here we will summarize the individual Rab40 proteins and their currently understood roles as they relate to actin dynamics and cell migration.

## 5 The role of individual Rab40 family members in actin regulation and cell migration

### 5.1 Rab40A

The least studied member of the Rab40 family, Rab40A has been implicated in regulating migration and the actin cytoskeleton through the control of focal adhesion sites. By binding to Cullin5, Rab40A is able to ubiquitinate the small GTPase RhoU, resulting in RhoU degradation. Interestingly, in a kinase independent manner, binding of P21 activated kinase 4 (PAK4) blocks RhoU from being ubiquitinated. In focal adhesions, when RhoU is saved from degradation by binding PAK4, RhoU functions to promote the phosphorylation of paxillin at residue Ser272. This specific paxillin phosphorylation ultimately promotes focal adhesion disassembly and turnover, causing efficient cell migration. Thus, Rab40A can modulate cell migration through regulating focal adhesion turnover (Figure 3B) (Dart et al., 2015).

### 5.2 Rab40AL

Despite its sequence similarity to Rab40A, Rab40AL has not yet been studied in the context of focal adhesions. Accordingly, Rab40AL has not been linked to the actin cytoskeleton and has only indirectly been linked to cell migration. In non-small cell lung cancer cells, microarray-based analysis found that *Rab40AL* is one of multiple genes whose expression has been elevated more than five-fold as compared to normal expression levels upon exposure to nitric oxide. In lung cancer, nitric oxide exposure often leads to increased invasion and metastasis of cancer cells. Thus, while indirect, these data suggests that Rab40AL may be a positive regulator of lung cancer cell migration (Maiuthed et al., 2020). Previous work has also shown that Rab40AL loss-of-function mutation may contribute to Martin-Probst Syndrome (MPS). MPS is a multiorgan developmental disorder causing phenotypes such as cognitive impairment, hearing loss, craniofacial dysmorphism, and short stature; phenotypes which may result from improper cell migration during development. The Rab40AL D59G mutation was originally identified in two male patients with MPS and was further found in three other male subjects with MPS symptoms (Jirair Krikor et al., 2012; Lee et al., 2014). Investigation found that the D59G mutation reduced Rab40AL protein level and altered Rab40AL cytoplasmic localization (Jirair Krikor et al., 2012). However, the role of the D59G Rab40AL mutation in causing MPS has been contested as multiple healthy individuals have also been found to carry the D59G Rab40AL mutation (Ołdak et al., 2014; Bianco et al., 2015; Ołdak et al., 2015).

### 5.3 Rab40B

Rab40B has been associated with a variety of metastatic cancer types, indicating that it has a role in dysregulated migration. In triple-negative breast cancer and esophageal adenocarcinoma, Rab40B has been found to be overexpressed. In non-small cell lung carcinoma samples, Rab40B was shown to be overexpressed specifically in areas of cancer cell invasion and metastasis (Liu et al., 2018; Zacharias et al., 2018; Li et al., 2023). Finally, study of Rab40B in patients with hepatocellular carcinoma and gastric cancer has shown that Rab40B expression is correlated with poor patient prognosis and cancer metastasis. Study of these cancer cell lines has shown that Rab40B overexpression is sufficient to increase cell proliferation, invasion, and migration (Li et al., 2015; Shi et al., 2020), suggesting that Rab40B plays an important role in regulating cancer cell metastasis.

Mechanistically, Rab40B has been identified as necessary for the trafficking of MMP 2 and 9 in vesicles to the invadopodia for secretion. In 3D migration assays, it was found that during MMP2/9 trafficking and secretion, Rab40B binds to tyrosine kinase substrate 5 (Tks5). Tks5 is a large scaffold known to bind invadopodia plasma membranes, and as such, through its interaction with Rab40B, Tks5 may act as a tether for MMP2/9 containing vesicles, targeting the vesicles to the extending invadopodia (Jacob et al., 2013; Jacob and Prekeris, 2015; Jacob et al., 2016). Interestingly, Rab40B interaction with Tks5 has been found to be independent of Rab40B-Cullin5 binding, indicating that Tks5 is not a target of Rab40B mediated ubiquitination (Figure 3B) (Linklater et al., 2021).

During migration, Rab40B has also been found to modulate the actin cytoskeleton. Through its function with Cullin5, Rab40B ubiquitinates the epithelial protein lost in neoplasia (EPLIN). Local ubiquitination of EPLIN at the lamellipodia results in EPLIN localization at stress fibers. Overexpression of the Rab40B SOCS 4A mutant results in increased EPLIN levels and EPLIN localization to both the stress fibers and the lamellipodia. Improper localization of EPLIN to the lamellipodia decreases actin ruffling and increases the number of stress fibers in the cell (Linklater et al., 2021). Rab40B also directly modulates actin and cell migration through its ubiquitination of Rap2. Rap2 has previously been found to promote cell migration and modulate the actin cytoskeleton. Interestingly, instead of regulating Rap2 degradation, Cullin5-Rab40B complex mediated mono-ubiquitination of Rap2 results in Rap2 activation and localization to the leading edge of a cell. Without Rab40B, Rap2 is trafficked to the lysosome for degradation (Duncan et al., 2022). Thus, Rab40B modulates the actin cytoskeleton and promotes cell migration through differential ubiquitylation of its substrates Rap2 and EPLIN (Figure 3B).

### 5.4 Rab40C

Rab40C is the most studied member of the Rab40 family. However, many studies involving Rab40C have not been related to cell migration and actin cytoskeleton dynamics, and so will not be discussed here. Similarly to Rab40B, *Rab40C* expression has been analyzed in a variety of cancer types. In prostate cancer and breast cancer, the *Rab40C* gene has been found to be hypermethylated. While this could point to changes in Rab40C protein levels, results analyzing *Rab40C* expression in these samples are negative (Geybels et al., 2015; Khakpour et al., 2017). Rab40C overexpression has been noted in gastric cancer and Lung Squamous Cell Carcinoma, while, in cases of osteosarcoma resulting in death, Rab40C levels are reduced (Rothzerg et al., 2021). The linking of Rab40C to a variety of cancer types indicates that dysregulation of *Rab40C* expression may contribute to cancer metastasis.

In non-cancerous conditions, Rab40C levels were found to be elevated during the migration phase of wound healing in immune cells (Mori et al., 2011). Furthermore, depletion of Rab40C in *Bactrocera dorsalis* (Oriental Fruit Fly) reduces reproductive success and the number of eggs laid by females, indicating that Rab40C may be involved in migration during the early development of fruit flies (Zheng et al., 2022).

Mechanistically, Rab40C has been shown to be essential for chemo-attractant based cell migration through ubiquitination and degradation of the Receptor for Activated C Kinase 1 (RACK1). RACK1 is known as a negative regulator of chemo-attractant induced cell migration (Day et al., 2018). Rab40C then functions to promote cell migration by reducing RACK1 levels (Figure 3B). Furthermore, Rab40C has been directly tied to modulation of the actin cytoskeleton by regulating focal adhesion sites. Loss of Rab40C increases focal adhesion number in the migrating cell. The Rab40C-Cullin5 complex ubiquitinates the ankyrin repeat domain 28 protein (ANKRD28) which is then targeted for degradation. ANKRD28 is a member of a protein phosphatase 6 complex (PP6) which, among other things, inhibits focal adhesion site formation (Han et al., 2022). Thus, Rab40C may regulate cell migration by preventing the accumulation of ANKRD28-PP6 at sites of focal adhesion formation (Figure 3B).

Interestingly, Rab40C is the main protein to be studied in the context of redundant function of the Rab40 family. In the context of focal adhesion sites, Rab40C is unique among the other Rab40 family members in regulating focal adhesion number (Han et al., 2022). However, similar to Rab40B, Rab40C has been found to also ubiquitinate EPLIN and also play a role in MMP secretion (Jacob et al., 2013; Linklater et al., 2021). As such, in relation to migration and the actin cytoskeleton, Rab40C seems to have both redundant and unique functions among the Rab40 family.

## 6 Summary and outstanding questions

The actin cytoskeleton forms a variety of structures which provide the basis for establishing the forces required for cell migration. While these structures are well described and have been reviewed previously, the regulation of these actin-based structures remains less understood. The Rho family of GTPases is canonically known as the main protein family involved in actin cytoskeleton regulation. However, the conflict between RhoA and Rac1 activity at the leading edge of the cell, combined with the plethora of ways and magnitudes to which the actin cytoskeleton can dynamically change, indicate that a variety of other mechanisms and pathways have evolved to regulate actin dynamics.

The Rab40 family of proteins has recently been shown to have roles influencing the actin cytoskeleton and cell migration. Rab40 family members exhibit unique evolutionary origins as they duplicated from *Rab18* and gained a SOCS box domain, making them the only known small monomeric GTPase that has been directly implicated in mediating protein ubiquitylation. During vertebrate evolution, *Rab40* underwent subsequent duplications, forming the four-protein family seen in higher primates. Why higher primates would need two extra members of the Rab40 family remains unclear. It is possible, however, that increased demands of actin cytoskeleton organization and migration during the development of increasingly complex vertebrate systems may have provided evolutionary pressures that led to the addition of two new members of Rab40 subfamily in higher primates.

All Rab40 family members stand apart from other Rab GTPases through the addition of a C-terminally located SOCS box domain, allowing them to bind to Cullin5 and function as part of an E3 ubiquitin ligase complex. While many roles of the Rab40 family members in regulating actin dynamics have been found to be a result of Rab40-mediated ubiquitination of their specific substrates, some Rab40 functions, such as the involvement of Rab40B in MMP secretion, appear to be independent of Rab40 mediated ubiquitination. This suggests that Rab40 family members evolved to have multiple mechanisms of function throughout a cell, both functioning as membrane trafficking regulators (canonical function of Rab GTPases) and as E3 ligase adaptors that specify targets of ubiquitination. The potential dual functionality of Rab40 proteins provides an intriguing area for further study. Indeed, it is recently suggested that Rab40 regulates target protein ubiquitination at the specific subcellular domains, such as the lamellipodia of a migrating cell. Thus, during evolution, the Rab40 subfamily may have emerged as a means of targeting, tethering, and activating Cullin5 dependent E3 ligase complexes to specific cellular membranes.

Furthermore, Rab40B and Rab40C stand apart further from the rest of the Rab GTPases due to their unique glycine residue upstream of Switch-1, which is always a serine or threonine reside in other Rab proteins. The role of this glycine residue remains to be explored and raises questions regarding the regulation of Rab40B and Rab40C GTP/GDP binding, specifically calling into question the interaction of GEFs with Rab40B and Rab40C.

Another major remaining question is the potential redundancies of the different Rab40 isoforms. So far, studies have focused on individual family members and little work has been done to assess redundancy among these proteins. Recent work with Rab40B and Rab40C has begun to address this question, finding that both appear to have redundant and unique roles among the Rab40 family members. Thoroughly understanding these redundancies may provide unique evolutionary insight into duplication events occurring throughout vertebrate evolution.

Overall, the study of the Rab40 family of proteins remains relatively new. The Rab40 family remains unique among Rabs and has been shown to provide a level of control over cell migration and the actin cytoskeleton. How this regulation fits into the network of regulation established by Rho GTPases remain unknown, however, continued study of the Rab40 family can only serve to further our understanding of the actin cytoskeleton, cell migration, and the complex mechanisms which regulate them.
